# Detecting faking-good response style in personality questionnaires with four choice alternatives

**DOI:** 10.1007/s00426-020-01473-3

**Published:** 2021-01-16

**Authors:** Merylin Monaro, Cristina Mazza, Marco Colasanti, Stefano Ferracuti, Graziella Orrù, Alberto di Domenico, Giuseppe Sartori, Paolo Roma

**Affiliations:** 1 Department of General Psychology, University of Padova, Padua, Italy; 2 Department of Neuroscience, Imaging and Clinical Sciences, University “G.d’Annunzio”, Chieti, Pescara Italy; 3 Department of Human Neuroscience, Sapienza University of Rome, Rome, Italy; 4 Department of Surgical, Medical, Molecular and Critical Area Pathology, University of Pisa, Pisa, Italy; 5 Department of Psychological, Health and Territorial Sciences, University “G.d’Annunzio”, Chieti, Pescara Italy

## Abstract

Deliberate attempts to portray oneself in an unrealistic manner are commonly encountered in the administration of personality questionnaires. The main aim of the present study was to explore whether mouse tracking temporal indicators and machine learning models could improve the detection of subjects implementing a faking-good response style when answering personality inventories with four choice alternatives, with and without time pressure. A total of 120 volunteers were randomly assigned to one of four experimental groups and asked to respond to the Virtuous Responding (VR) validity scale of the PPI-R and the Positive Impression Management (PIM) validity scale of the PAI via a computer mouse. A mixed design was implemented, and predictive models were calculated. The results showed that, on the PIM scale, faking-good participants were significantly slower in responding than honest respondents. Relative to VR items, PIM items are shorter in length and feature no negations. Accordingly, the PIM scale was found to be more sensitive in distinguishing between honest and faking-good respondents, demonstrating high classification accuracy (80–83%).

**Supplementary Information:**

The online version contains supplementary material available at 10.1007/s00426-020-01473-3.

## Introduction

Personality questionnaires are the most popular tool used to measure personality for a variety of purposes, from pre-employment assessment to forensic evaluation (e.g., in the context of child custody hearings), (Burla et al., 2019; Mazza, Orrù, et al., 2019, Mazza, Monaro et al., 2019; Roma, Piccinni, & Ferracuti, 2016; Roma et al., 2013, 2014, 2019). However, the most favorable responses to items on these tests are often easily determined. For this reason, test-takers may decide, depending on their motivation, to distort their responses to achieve personal goals; such behavior is known as faking (Mazza, Orrù, et al., 2019; Sartori, Zangrossi, Orrù, & Monaro, 2017; Ziegler, MacCann, & Roberts, 2011). Faking-good, more specifically, is a behavior in which subjects present themselves in a favorable manner, endorsing desirable traits and rejecting undesirable ones. The general prevalence of faking-good is unknown; however, Baer and Miller (2002) estimated its rate to be approximately 30% for job applicants. Indeed, up to 63% of applicants admit to faking on personality tests (Dwight & Donovan, 2003); 50% admit to exaggerating positive qualities, while 60% admit to de-emphasizing negative traits (Donovan, Dwight, & Hurtz, 2003).

Most tests include validity scales designed to detect response bias (Paulhus, 2002)—otherwise known as the systematic tendency to answer items of a self-report test in a way that interferes with accurate self-presentation. However, these validity scales are often comprised of highly transparent items and are thus not always effective in detecting faking; therefore, some authors developed different indices, based on the best combination of scales, that could differentiate between honest respondents and fakers (Bosco et al. 2020; Martino et al. 2016), while other authors suggested that indirect behavioral measures could be accurate in detecting deception.

Starting in the early 1970s, Dunn, Lushene, & O’Neil (1972) suggested that response times (RTs) could assist in distinguishing fakers from honest respondents. The idea behind this theory is that the cognitive processes involved in lying differ from those involved in answering truthfully. Specifically, the literature indicates that lying requires more time, as it is cognitively more demanding than telling the truth; therefore, fakers typically record longer RTs (Foerster et al., 2013; Holden & Kroner, 1992; Mazza, Orrù et al., 2019, Mazza, Burla et al., 2019; McDaniel & Timm, 1990; Roma et al., 2018; Roma, Giromini et al., 2020, Roma, Mazza et al., 2020; Verschuere, 2018; Walczyk, Roper, Seemann & Humphrey, 2003). A meta-analysis indicated that honest and faking respondents show significantly different RTs when endorsing an item, but similar RTs when rejecting an item, suggesting that the type of answer could play a role in this regard (Maricuţoiu & Sârbescu, 2016). Moreover, there is evidence suggesting that the introduction of a false alibi may invalidate these effects, facilitating dishonest responses and making honest retrieval more effortful (Foerster 2017).

Another line of research suggests that time pressure (i.e., limited time available to answer), leads to less ethical decision making and responses that emphasize socially approved traits and behavior (Gunia et al., 2012; Khorramdel & Kubinger, 2006; Neubauer & Malle, 1997; Shalvi, Eldar, & Bereby-Meyer, 2012, 2013; Sutherland, 1964). In detail, when respondents are presented with an immediate choice or have limited time available to answer, they tend to lie more frequently; this makes their faking more easily detectable. In contrast, when participants have sufficient time to reflect, they tend to choose their answers more cautiously and moderate their faking behavior. Roma et al. (2018) found support for this idea in research using the Minnesota Multiphasic Personality Inventory-2 Restructured Form (MMPI-2-RF), (Ben-Porath & Tellegen, 2008; Tellegen & Ben-Porath, 2011): in a sample of 135 male volunteers, participants instructed to fake under time pressure obtained significantly higher T-scores^1^ on the L-r and K-r scales when compared to fakers in the unspeeded condition ($\eta_{\text{p}}^2$ = 0.243). These findings were later confirmed by a study (Roma, Mazza et al., 2020) using the MMPI-2 underreporting scales (L, K, S), (Butcher, 2001; Hathaway, McKinley, & Committee, 1989): faking-good respondents in the speeded condition scored higher T-scores on the L and K scales than did faking-good respondents without time pressure (MMPI-2 L scale $\eta_{\text{p}}^2$ = 0.481; MMPI-2 K scale $\eta_{\text{p}}^2$ = 0.457; MMPI-2 S scale $\eta_{\text{p}}^2$ = 0.011). Furthermore, the latter study also highlighted that the effect of time pressure was noticeable only in the faking condition, while honest respondents remained honest in both conditions; this suggests that speeded answering may not always trigger faking. Finally, a recent analysis employing machine learning (ML) models trained on behavioral features (e.g., RT, time pressure) to identify fakers in self-report questionnaires indicated that time pressure was the most reliable method for identifying faking-good behavior Mazza et al. (2019). However, the effect of speeded tests on RT is debated: a recent meta-analysis (Verschuere, 2018) indicated that cognitive load (e.g., time pressure) could generate higher RTs in honest subjects, thereby decreasing the RT difference between faking and honest respondents by impeding respondents’ ability to quickly tell the truth (*g* = − 0.184).

In recent years, research has evaluated the efficacy of using mouse dynamics to detect deception. Specifically, mouse tracking records the cursor’s position, enabling researchers to follow mouse trajectories from the beginning to the end of a movement (Freeman & Ambady, 2010). This procedure has yielded promising results in lie detection studies, highlighting as trajectories data can be a powerful and rich source of cues to detect liars.

One of the pioneering studies in this field recorded the hands dynamics through a Nintendo Wii controller, while the subjects were engaged in an instructed lying task (Duran, Dale, & McNamara, 2010). The analysis of motor trajectories revealed that instructed lies could be distinguished from truthful responses according to the motor onset time, the overall response time, the trajectory, the velocity and the acceleration of the movement. Similarly, it has been shown that the analysis of movement trajectories of participants engaged in mouse-tracking (Pfister et al., 2016) and finger-tracking paradigms (Wirth et al., 2016) can reveal the on-going conflicts caused by a voluntary and deliberate rule violation. More recently, a series of studies conducted by Monaro et al. have suggested that, when completing autobiographical inventories, honest respondents follow a direct trajectory from the starting point to the desired answer, whereas fakers show larger and less straight trajectories that initially point towards the actual autobiographical information and then switch in the direction of the alternative (Monaro, Gamberini, and Sartori, 2017; Monaro et al., 2018). Other studies have demonstrated that it is possible to identify patients simulating symptoms of depression and amnesia with accuracies ranging from 80 to 90% by analyzing their mouse dynamics when responding to questions about their symptoms (Monaro et al., 2018, Monaro, Gamberini, et al., 2018; Zago et al., 2019). A more recent study (Mazza et al., 2020) highlighted that honest respondents are faster than fakers in moving along the *x*-axis when responding to the MMPI-2 underreporting scales (S, K, L); they are also faster in moving along the *y*-axis when responding to the K scale and Psychopatic Personality Inventory Revised (PPI-R) VR scale. Furthermore, this study found significantly larger RTs and MD-times (i.e., maximum deviation time, or the time to reach with the mouse the point of maximum distance between the actual and the idealized trajectory) in the faking-good condition compared to the honest test-takers, but only for the L scale.

While mouse tracking software enables researchers to also record RTs, it is worth noting that these RTs are not exactly equivalent to the simple RTs used in the aforementioned studies (Foerster et al., 2013; Holden & Kroner, 1992; Mazza, Orrù et al., 2019, Mazza, Burla et al., 2019; McDaniel & Timm 1990; Roma et al., 2018; Roma, Giromini et al., 2020, Roma, Mazza et al., 2020; Verschuere, 2018; Walczyk, Roper, Seemann & Humphrey, 2003), since they include both cognitive and motor components. Moreover, mouse dynamics have nonetheless proven useful in lie detection research as they have been used to collect data on a large number of features (e.g., initiation time, time to reach the point of maximum mouse deviation, etc.) that can be used as predictors of deception.

To date, studies investigating the relationship between faking and behavioral indicators have largely used tests with dichotomous choice alternatives (i.e., true vs. false). However, many personality inventories adopt Likert scales as a response mode (e.g., strongly agree, agree, moderately agree, disagree, strongly disagree). For this reason, the present study used the underreporting scales of the Personality Assessment Inventory (PAI) and the Psychopathic Personality Inventory-Revised (PPI-r), which were designed to detect overly favorable self-presentations on items with four choice alternatives. To the best of our knowledge, this was the first study on faking-good using exclusively multiple-choice items, specifically with four alternatives. While the literature on this topic is scarce, it indicates that subjects take longer to react to four stimuli than to two (Garner, 1962; Kiesler, 1966); therefore, the number of response alternatives may affect RT and mouse dynamics and interact with the effect of deception and time pressure. Williams, Bott, & Lewis (2013) reported that increasing the number of possible lie responses—from one to two or three—leads to a greater lying latency effect in subjects.

The aim of the present study was to evaluate the usefulness of T-scores on underreporting scales and behavioral features (i.e., RT and mouse dynamics) in detecting faking-good behavior when items have four, rather than two, choice alternatives. Building on previous findings (Mazza et al. 2020), the hypotheses were as follows: H1) Mouse movements (temporally described by RT, MD-time, velx and vely) would be slower in the faking-good condition relative to the honest condition.

H2) T-scores on the PPI-R VR scale and the PAI PIM would be higher in the faking-good speeded condition relative to the faking-good unspeeded condition; T-scores of honest respondents would not show any significant differences between speeded and unspeeded conditions.

Finally, similarly to previous studies (Monaro et al. 2018, Monaro, Gamberini, et al. 2018; Zago et al. 2019), here we assess the accuracy of the above-mentioned measures (T-scores and mouse tracking temporal features) in predicting whether a subject is having a faking-good behavior or not. Focusing on prediction rather than explanation when data analysis is performed is a recent and increasingly widespread trend in different scientific fields (Yarkoni & Westfall 2017), including a wide range of human research areas, like smart applications (Spolaor et al., 2018), genetics (Navarin & Costa, 2017), clinical medicine (Obermeyer & Emanuel, 2016) and clinical psychology (Monaro et al., 2018, Monaro, Gamberini, et al. 2018). This trend is becoming increasingly popular also thanks to the exponential growth of Machine learning (ML), a branch of artificial intelligence that deals with training algorithms to automatically learn information from a set of data and make predictions on a completely new set of unseen data without being explicitly programmed. ML techniques have already been used in behavioral science to predict human malicious behaviors, for example to identify people who declared false identities (Monaro, Gamberini, and Sartori 2017), who simulate depression (Monaro et al. 2018, Monaro, Gamberini, et al. 2018) or amnesia (Zago et al. 2019). From an applicative point of view, one of the main advantages of using ML is that it makes it possible to make predictions at the individual level, while traditional statistical methods just make inferences on the group level (Orrù et al. 2020). In other words, ML algorithms provide a useful and automatic tool to identify people who produce malicious behaviors in a clinical setting. In this research, ML algorithms are trained to investigate the accuracy of T-score and temporal mouse tracking variables in identifying faking-good respondents to the PPI-R VR scale and PAI PIM scale.

## Materials and methods

### Participants

A total of 120 young adults voluntarily participated in the study. The only inclusion criterion was that participants needed to be able to read questions on a computer monitor, understand the meaning of those questions, and answer the questions via a computer mouse. The sample was comprised of males (50%) and females (50%) aged 18–30 years (*M* = 22.73; SD 2.84) who were non-psychology graduates (i.e., their degree was in a discipline other than psychology) and Caucasian. Participants were randomly assigned to one of four experimental groups defined by various combinations of the manipulated factors of instructions (honest [H] vs. faking-good [FG]) and time pressure (speeded [S] vs. unspeeded [U]): (a) group 1 (*N* = 30) (*M*_age_ = 23.53; SD 2.70) had honest–faking-good unspeeded conditions (H-FG/U); (b) group 2 (*N* = 30) (*M*_age_ = 21.97; SD 2.57) had faking-good–honest unspeeded conditions (FG-H/U); (c) group 3 (*N* = 30) (*M*_age_ = 22.67; SD 2.91) had honest–faking-good speeded conditions (H-FG/S); and (d) group 4 (*N* = 30) (*M*_age_ = 22.77; SD 3.08) had faking-good–honest speeded conditions (FG–H/S). Using G*Power software, it has been calculated that using a repeated measures ANOVA test, with between-within interactions, a statistical power of (1 − β) = 0.95 may be achieved with a sample size of 70, given a number of groups of 2, a number of repeated measurements of 2, a significance level (*α*) of 0.01 and a large effect size (*f*) of 0.26 (Faul, Erdfelder, Lang & Buchner, 2007). In the present study, no statistically significant differences were observed with respect to age.

All participants provided informed consent before the research began. They did not receive any compensation for their participation. The experimental procedure was approved by the local ethics committee (Board of the Department of Human Neuroscience, Faculty of Medicine and Dentistry, Sapienza University of Rome), in accordance with the Declaration of Helsinki.

### Materials

#### PAI positive impression management (PIM) validity scale

The Personality Assessment Inventory (PAI), (Morey, 1991), is a 22-scale self-report measure of personality and psychopathology consisting of 344 items. Test-takers respond to each item on a four-point scale (true vs. mostly true vs. mostly false vs. false). In particular, the present study focused on the Positive Impression Management (PIM) validity scale (9 items; e.g., “I don’t take criticism very well”), which assesses the degree to which respondents present themselves in a favorable fashion or an overly positive manner. The Italian version of the PAI was edited by Zennaro et al. (2015).

#### PPI-R Virtuous Responding (VR) validity scale

The Psychopathic Personality Inventory-Revised (PPI-R), (Lilienfeld & Widows 2005), is a 154-item personality questionnaire articulated in 8 subscales that assess traits associated with psychopathy. Test-takers respond to each item on a four-point scale (true vs. mostly true vs. mostly false vs. false). The present study used the PPI-R Virtuous Responding (VR) validity scale, which is comprised of 13 items (e.g., “I’ve never desired to hurt someone”) and designed to detect underreporting. The Italian version of the PPI-R was edited by La Marca et al. (2008).

### Research Design

The present study featured a mixed design with two manipulated factors: instructions (within subject factor, H vs. FG) and time pressure (between subject factor, U vs. S). As described above, participants were randomly assigned to one of four experimental groups that combined these factors: H–FG/U, FG–H/U, H–FG/S, and FG–H/S. In the first group (H–FG/U), participants were asked to complete the tests (PPI-R VR scale and PAI PIM scale) without time pressure. They were initially instructed to respond honestly (1a) and then to fake good (1b). Specifically, the instructions were as follows (see also Roma et al., 2018; Mazza et al., 2020):

1a: We are interested in some characteristics of your personality. We want you to take this test in a totally sincere fashion. Pay attention, because the questionnaire contains features designed to detect faking. After reading each item you should take all the time you need to respond in the best way.
1b: You just completed the test honestly. Now imagine that you are applying for a desired job. In this situation, it would be to your advantage to appear as if you were completely normal and psychologically healthy. Stated differently, we want you to take this test and deliberately fake good. Pay attention, because the questionnaire contains features designed to detect faking, and your intent is to respond in a way that your deception cannot be detected. After reading each item you should take all the time you need to respond in the best way, according to this instruction.

In the second group (FG–H/U), participants completed the test without time pressure, first with the instruction to fake good (2a) and then with the instruction to respond honestly (2b). Specifically, the instructions were as follows:

2a: We are interested in some characteristics of your personality. Imagine you are applying for a desired job. In this situation, it would be to your advantage to appear as if you were completely normal and psychologically healthy. Stated differently, we want you to take this test and deliberately fake good. Pay attention, because the questionnaire contains features designed to detect faking, and your intent is to respond in a way that your deception cannot be detected. After reading each item you should take all the time you need to respond in the best way, according to this instruction.
2b: You just completed the test dishonestly. Now, we are interested in some real characteristics of your personality. We want you to take this test in a totally sincere fashion. Pay attention, because the questionnaire contains features designed to detect faking. After reading each item you should take all the time you need to respond in the best way.

In the third group (H–FG/S), participants completed the test with time pressure, first with the instruction to respond honestly (3a) and then with the instruction to fake good (3b). Specifically, the instructions were as follows:

3a: We are interested in some characteristics of your personality. We want you to take this test in a totally honest fashion. Pay attention, because the questionnaire contains features designed to detect faking. After reading each item you should respond as quickly as possible. Short response time is an important factor in this test.
3b: You just completed the test honestly. Now imagine that you are applying for a desired job. In this situation it would be to your advantage to appear as if you were completely normal and psychologically healthy. Stated differently, we want you to take this test and deliberately fake good. Pay attention, because the questionnaire contains features designed to detect faking, and your intent is to respond in a way that your deception cannot be detected. After reading each item you should respond as quickly as possible. Short response time is an important factor in this test.

Finally, in the fourth group (FG–H/S), participants completed the test with time pressure, first with the instruction to fake good (4a) and then with the instruction to respond honestly (4b). Specifically, the instructions were as follows:

4a: We are interested in some characteristics of your personality. Imagine you are applying for a desired job. In this situation it would be to your advantage to appear as if you were completely normal and psychologically healthy. Stated differently, we want you to take this test and deliberately fake good. Pay attention, because the questionnaire contains features designed to detect faking, and your intent is to respond in a way that your deception cannot be detected. After reading each item you should respond as quickly as possible. Short response time is an important factor in this test.
4b: You just completed the test dishonestly. Now, we are interested in some real characteristics of your personality. We want you to take this test in a totally honest fashion. Pay attention, because the questionnaire contains features designed to detect faking. After reading each item you should respond as quickly as possible. Short response time is an important factor in this test.

### Procedure and stimuli

Participants completed the experimental task individually in a quiet room within the Department of Human Neuroscience at the Sapienza University of Rome. The task was run on a 15-in. laptop using a Microsoft Windows operating system, with participants sitting approximately 60 cm from the screen. Following their initial reception, proceeded according to these sequential steps: (a) they provided their informed consent, (b) they completed a demographic questionnaire, (c) they were assigned to one of the four experimental groups, (d) they completed the experimental task (scripts PIM and VR) following their respective group’s first instructions (instructions 1a, 2a, 3a, 4a, abovementioned), (5) they viewed an unrelated short video, and (6) they completed the experimental task (scripts PIM and VR) following their respective group’s second instructions (instructions 1b, 2b, 3b, 4b, abovementioned).

**Fig. 1:**
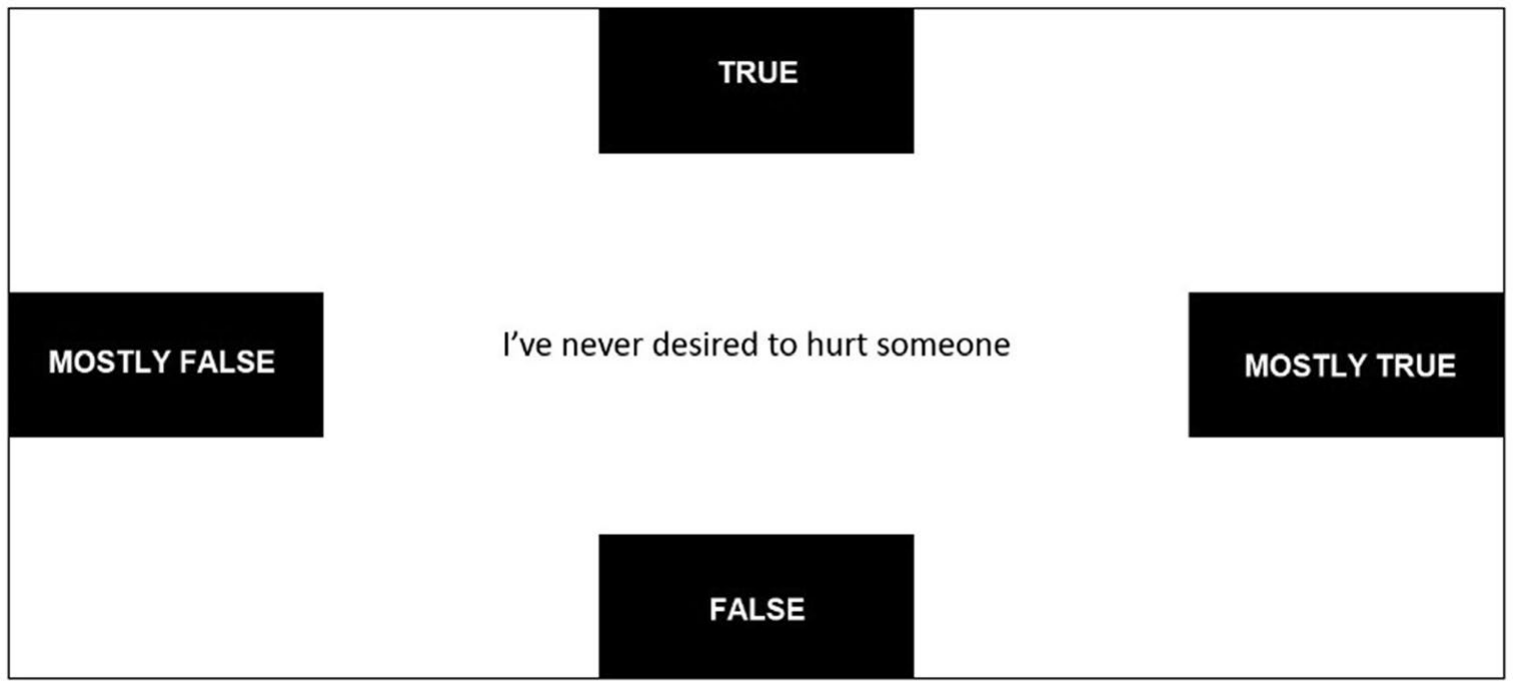
Screenshot of the experimental task as it appeared to participants. Note. The START button was in the central part of the screen, in the same location as the item displayed in this figure. After START was pressed, the item text appeared

The experimental task was programmed using the MouseTracker Software (Freeman & Ambady, 2010). The task consisted of the 22 stimuli (i.e., items) belonging to the PAI PIM scale and the PPI-R VR scale. The presentation order of the stimuli reflected the item appearance order of the original scales. Both the VR and PIM items were preceded by one training question. Stimuli were presented in the central display of the computer screen. Participants had to initiate the presentation of each question by clicking (with the mouse) a START button located in the central part of the screen, then they had to respond to each question by choosing one of four alternative response buttons (TRUE vs. MOSTLY TRUE vs. MOSTLY FALSE vs. FALSE). Response buttons were equidistant from the item text and the mouse starting point (START button). According to previous literature (Monaro, Gamberini, and Sartori, 2017) the position of the response labels remained fix during the entire experiment. Indeed, it has been shown that keeping the labels fixed on the screen would not lead to response biases (Monaro, Gamberini, and Sartori, 2017). An example of the computer screen as it appeared to participants during the experimental task is displayed in Fig. 1.

### Collected measures

The software recorded all responses given by all participants to each item. T-scores were calculated separately for the VR and PIM scales, according to the Italian validations of the measures. During the experimental task, the MouseTracker software also recorded the temporal features (in milliseconds) of each response. Each response trajectory was described in the following features:

- Response time (RT): the time between the appearance of the item and the mouse click on the response button.
- Maximum deviation time (MD-time): the time taken by the respondent to reach (with the mouse) the point of maximum deviation—the maximum perpendicular distance (MD) between the actual and the idealized trajectory; the idealized trajectory represents the virtual straight line connecting the starting point to the endpoint (the response button). Thus, the higher the MD, the more the trajectory deviated toward the unselected alternatives. It should be noted that in four-choices paradigms, the MouseTracker software allows to take all trials and remap them vertically to one response (e.g., true) and be rotated such that the hypothetical distractor (the unselected alternative) is located at another response (e.g., mostly true).
- Velocity along the *x*-axis (vel_x_): the average speed of mouse movement along the *x*-axis between two-time frames. It is worth noting that the MouseTracker software normalizes each mouse trajectory in 101 temporal frames using linear interpolation; in this way, each trajectory has 101 temporal frames and each time frame has corresponding *x* and *y* coordinates. For example, the coordinate (*x*_1_,*y*_1_) corresponds to the position of the mouse on the *x*-axis and *y*-axis at time frame 1 (Freeman 2015).
- Velocity along the *y*-axis (vel_y_): the average speed of mouse movement along the *y*-axis between two-time frames.

Although the MouseTracker Software is capable of collecting a larger number of features (i.e., maximum perpendicular distance between the actual and idealized trajectory, area between the actual and idealized trajectory, number of flips on the *x* and *y* axes), previous studies have demonstrated that only temporal features are useful predictors of deception when responding to the underreporting scales of a personality questionnaire (Mazza et al., 2020) or, more generally, when responding to complex questions (Monaro, Gamberini, and Sartori, 2018). For this reason, the present study analyzed only temporal features. Finally, for each temporal feature (RT, MD-time, vel_x_, vel_y_), the average response value for each scale (VR, PIM) was computed, generating eight variables (RT PIM, RT VR, MD-time PIM, MD-time VR, vel_x_ PIM, vel_x_ VR, vel_y_ PIM, vel_y_ VR). Thus, including PIM and VR T-scores, a total of ten variables were included and analyzed.

## Results

### Univariate analyses of variance

To test the difference between the four experimental conditions (instructions: H vs. FG; time pressure: U vs. S), a mixed ANOVA was run for each investigated variable (RT PIM, RT VR, MD-time PIM, MD-time VR, vel_x_ PIM, vel_x_ VR, vel_y_ PIM, vel_y_ VR, PIM T-score, VR T-score). To address the problem of multiple testing, the Bonferroni correction was applied, dividing the *p* value by the number of tested variables for each scale (*n* = 5) and setting the significance level to 0.01 (Shaffer, 1995). Analyses were computed using the “ez” package in the R software.

Tables 1 and 2 report the results of the ANOVA for the VR and PIM scale, respectively.

**Table 1:** Results of the ANOVA mixed models computed for the VR scale

Variable	Effect	*F*	*p* value	$\eta_{\text{G}}^2$
*T*-score VR	Instructions*	*F*_(1,118)_ = 351.017	3.696e^−37^	0.495 (large)
	Time pressure	*F*_(1,118)_ = 0.866	0.354	< 0.02
	Instructions × time pressure	*F*_(1,118)_ = 3.951	0.049	< 0.02
RT VR	Instructions	*F*_(1,118)_ = 3.815	0.053	< 0.02
	Time pressure*	*F*_(1,118)_ = 29.897	2.585e^−07^	0.170 (medium)
	Instructions × time pressure	*F*_(1,118)_ = 5.670	0.019	< 0.02
MD-time VR	Instructions	*F*_(1,118)_ = 1.022	0.314	< 0.02
	Time pressure*	*F*_(1,118)_ = 14.566	2.171e^−04^	0.091 (small)
	Instructions × time pressure	*F*_(1,118)_ = 5.531	0.020	< 0.02
vel_x_ VR	Instructions	*F*_(1,118)_ = 3.301	0.072	< 0.02
	Time pressure	*F*_(1,118)_ = 0.221	0.639	< 0.02
	Instructions × time pressure	*F*_(1,118)_ = 0.747	0.389	< 0.02
vel_y_ VR	Instructions	*F*_(1,118)_ = 1.435	0.233	< 0.02
	Time pressure	*F*_(1,118)_ = 5.344	0.023	0.022 (small)
	Instructions × time pressure	*F*_(1,118)_ = 4.628	0.033	< 0.02

Statistically significant effects (*p* < 0.01) are marked (*). The final column reports the effect size (generalized eta squared, $\eta_{\text{G}}^2$). With respect to magnitude, $\eta_{\text{G}}^2$ = 0.02 was considered indicative of a small effect, $\eta_{\text{G}}^2$ = 0.13 of a medium effect, and $\eta_{\text{G}}^2$ = 0.26 of a large effect (Cohen 1988)

**Table 2:** Results of the ANOVA mixed models computed for the PIM scale

Variable	Effect	*F*	*p* value	$\eta_{\text{G}}^2$
T-score PIM	Instructions*	*F*_(1,118)_ = 321.667	1.692e^−35^	0.481 (large)
	Time pressure	*F*_(1,118)_ = 1.850	0.176	< 0.02
	Instructions X time pressure	*F*_(1,118)_ = 2.013	0.159	< 0.02
RT PIM	Instructions*	*F*_(1,118)_ = 11.538	9.29 e^−04^	0.027 (small)
	Time pressure*	*F*_(1,118)_ = 15.882	1.171e^−04^	0.087 (small)
	Instructions X time pressure	*F*_(1,118)_ = 3.621	0.059	< 0.02
MD-time PIM	Instructions	*F*_(1,118)_ = 5.679	0.019	< 0.02
	Time pressure*	*F*_(1,118)_ = 10.163	1.835e^−03^	0.054 (small)
	Instructions X time pressure	*F*_(1,118)_ = 2.532	0.114	< 0.02
vel_x_ PIM	Instructions*	*F*_(1,118)_ = 37.537	1.218e^−08^	0.111 (small)
	Time pressure	*F*_(1,118)_ = 0.534	0.466	< 0.02
	Instructions X time pressure	*F*_(1,118)_ = 0.030	0.863	< 0.02
vel_y_ PIM	Instructions*	*F*_(1,118)_ = 239.391	3.597e^−30^	0.438 (large)
	Time pressure	*F*_(1,118)_ = 0.166	0.684	< 0.02
	Instructions X time pressure	*F*_(1,118)_ = 0.656	0.420	< 0.02

Statistically significant effects (*p* < 0.01) are marked (*). The final column reports the effect size (generalized eta squared, $\eta_{\text{G}}^2$). With respect to magnitude, $\eta_{\text{G}}^2$ = 0.02 was considered indicative of a small effect, $\eta_{\text{G}}^2$ = 0.13 of a medium effect, and $\eta_{\text{G}}^2$ = 0.26 of a large effect (Cohen 1988)

#### VR scale

A significant effect was found for instructions on *T*-score. However, no main effect was found for instructions on temporal variables (RT, MD-time, vel_x_, vel_y_). Moreover, there was a significant effect of time pressure on RT and MD-time, but no main effect of time pressure on *T*-score, vel_x_, or vel_y_. Finally, no statistically significant results were generated by the interaction between time pressure and instructions.

In short, faking-good respondents obtained significantly higher T-scores (FG: *M* = 69.08, SD 10.46) on the VR scale relative to honest respondents (H: *M* = 48.74, SD 10.34). Participants under temporal pressure were significantly faster than respondents in the unspeeded condition in terms of RT (S: *M* = 4877.67, SD 1347.47; US: *M* = 6771.71, SD 2669.26) and MD-time (S: *M* = 3016.04, SD 983.29; US: *M* = 4037.22, SD 2090.52), independent of whether they were honest or faking-good.

#### PIM scale

A significant effect was found for instructions on *T*-score. The ANOVA also uncovered a main effect for instructions on all temporal variables (RT, vel_x_ and vel_y_), except for MD-time (although the *p* value was very close to significance, *p* = 0.019). Moreover, RT and MD-time significantly suffered from the main effect of time pressure. The analyses did not reveal any significant effect of time pressure on *T*-score, vel_x_, or vel_y_. The interaction between time pressure and instructions did not show any statistically significant result for any of the considered variables.

To summarize, as for the VR scale, on the PIM scale fakers achieved significantly higher *T*-scores (FG: *M* = 59.22, SD 9.95) than honest respondents (H: *M* = 40.99, SD 9.20). Also similar to the VR scale findings, PIM respondents in the speeded condition were faster than unspeeded subjects in terms of RT (S: *M* = 4460.40, SD 1365.43; US: *M* = 5693.08, SD 2535.63) and MD-time (S: *M* = 2758.22, SD 1041.53; US: *M* = 3404.05, SD 1638.80), independent of instructions. However, differently from the VR scale, on the PIM scale, faking-good participants achieved significantly slower RTs than honest respondents (FG: *M* = 5411.06, SD 2458.87; H: *M* = 4742.42, SD 1669.88); they were also significantly slower on average mouse speed along the *x*-axis (FG: *M* = − 0.0005, SD 0.0017; H: *M* = 0.0006, SD 0.0014) and *y*-axis (FG: *M* = − 0.0021, SD 0.0019; H: *M* = 0.0012, *SD* 0.0017).

Finally, to exclude an effect due to the order of presentation of the within subject factor (instructions H vs. FG), an independent sample *t* test was run comparing participants who took H condition first and then FG condition with participants who took FG condition firstly and H condition as the second one. Again, the Bonferroni correction was applied, setting the significance level to 0.01. The results excluded the presence of a sequence effect for all the investigated variables (RT PIM: *t*_(238)_ = − 1.454, *p* = 0.147; RT VR: *t*_(238)_ = − 1.309, *p* = 0.192; MD-time PIM: *t*_(238)_ = − 0.023, *p* = 0.982; MD-time VR: *t*_(238)_ = − 1.199, *p* = 0.232; vel_x_ PIM: t_(238)_ = 2.127, *p* = 0.034, vel_x_ VR: *t*_(238)_ = 0.491, *p* = 0.624; vel_y_ PIM: *t*_(238)_ = 0.770, *p* = 0.442; vel_y_ VR: *t*_(238)_ = 0.241, *p* = 0.810; PIM *T*-score: *t*_(238)_ = − 1.454, *p* = 0.147; VR *T*-score: *t*_(238)_ = − 1.788, *p* = 0.075).

### Predictive models

In the present study, ML analyses were run through the data mining software WEKA 3.9 (Hall et al., 2009). First, feature selection was performed using a correlation-based feature selector (CFS), with a “greedy stepwise” search method. The CFS algorithm is a simple filter algorithm that ranks the feature subsets according to a correlation based heuristic evaluation function (Hall, 1999). The bias of the evaluation function is toward subsets that contain features that are highly correlated with the class (in this case, FG vs. H) and uncorrelated with each other. Features with low correlation with the class are ignored, because they are irrelevant. Features that are highly correlated with one or more of the remaining features are screened out as they are redundant. This procedure aimed at removing redundant and irrelevant features and thereby increasing model generalization by reducing overfitting and removing noise from data.

Following this, model accuracy was evaluated using a tenfold cross-validation procedure (Kohavi, 1995). The *k*-fold cross validation consisted of randomly and repeatedly splitting the entire sample into parts: the training set and the validation set. This resampling procedure seeks to reduce variance in the model performance estimation with respect to using a single training set and a single validation set, reducing model overfitting (Kohavi, 1995). In the present work, the sample of 120 participants who performed the task twice (FG vs. H) was partitioned into *k* = 10 equal-size subsamples (10 folds of 12 participants who performed 2 tasks). Of the ten subsamples, nine were used to train the model and the remaining one was used to validate it. This process was repeated ten times, so each of the ten folds was used just once as a validation set. Finally, the average of the results obtained from the ten folds gave the estimation of the validation accuracy.

#### Models including both VR and PIM scales

With all ten variables (RT PIM, RT VR, MD-time PIM, MD-time VR, vel_x_ PIM, vel_x_ VR, vel_y_ PIM, vel_y_ VR, T-score PIM, *T*-score VR) included in the feature selection process, the CFS algorithm selected the following: *T*-score PIM (*r*_pb_ = 0.69), *T*-score VR (*r*_pb_ = 0.70), vel_x_ PIM (*r*_pb_ = 0.33), vel_y_ PIM (*r*_pb_ = 0.66). Note that *r*_pb_ indicates the value of the point-biserial correlation between the feature and the independent variable (FG vs. H).

Five ML algorithms—logistic (le Cessie & van Houwelingen, 1992), support vector machine (SVM), (Keerthi et al., 2001), naïve Bayes (John & Langley, 1995), random forest (Breiman, 2001) and logistic model tree (LMT), (Landwehr, Hall, & Frank, 2005) algorithms—were trained on the four selected variables using the tenfold cross-validation technique. The five classifiers were selected according to previous relevant studies (Mazza et al., 2020; Mazza, Orrù et al., 2019; Mazza, Burla et al., 2019) to facilitate the comparison of the results across different experiments. Moreover, they are representative of different underlying classification strategies to limit the possibility that the results would depend on the specific assumptions of each algorithm and to ensure that classification accuracy would be stable across classifiers. The parameters of the ML classifiers were those automatically chosen by the software WEKA 3.9 to run these algorithms (more details are reported in supplementary information).

Table 3 reports the accuracy, recall, precision, and *F*-score for each model. Precision (also known as positive predictive value) is the fraction of true positives among the retrieved instances (true positive + false positive), while recall (also called sensitivity or true positive rate) is the proportion of true positives that are correctly identified as such. The *F*-score is a measure of a test’s accuracy obtained by computing the harmonic mean of the precision and recall; it reaches its best value at 1. The results demonstrated that all classifiers had 85–86% accuracy in their ability to detect faking-good respondents.

**Table 3:** Results from the four ML classification models

ML classifier	Accuracy (%)	Precision	Recall	*F*-score
Logistic	85	0.852	0.850	0.850
SVM	85.42	0.863	0.854	0.853
Naïve Bayes	86.67	0.869	0.867	0.866
Random forest	85.83	0.858	0.858	0.858
LMT	85	0.852	0.850	0.850

For each classifier, the following metrics obtained from the tenfold cross-validation procedure are reported: validation accuracy, precision, recall, and F-score

#### VR scale model vs. PIM scale model

The univariate analyses of variance found that honest and faking-good respondents differed in their *T*-scores on both the VR and PIM scales, but only differed in their temporal mouse features on the PIM scale. The feature selection confirmed that the most relevant variables in detecting faking-good respondents on these scales were VR *T*-score and PIM *T*-score, as well as the temporal variables related to mouse velocity along the *x* and *y* axes on the PIM scale. In other words, temporal features distinguished honest from faking-good respondents only on the PIM scale (in that faking-good respondents were slower to reply than honest respondents only on this scale). To quantify this observation in terms of classification accuracy, we ran two sets of ML models that were trained, respectively, on the temporal features of the PIM scale (RT PIM, MD-time PIM, vel_x_ PIM, vel_y_ PIM) and the temporal features of the VR scale (RT VR, MD-time VR, vel_x_ VR, vel_y_ VR). Table 4 reports the classification results of the tenfold cross-validation. While classification accuracy based on PIM temporal features ranged from 76 to 82%, that of VR temporal features was only slightly above chance (55–57%). Moreover, as regards the PIM scale, the classification results highlighted that the temporal features of mouse trajectories, when used as predictors, achieved similar classification accuracies as T-scores (see Table 5).

**Table 4:** Results from four ML classification models trained on the temporal features of the PIM and VR scales, separately

Scale	ML classifier	Accuracy (%)	Precision	Recall	*F*-score
VR	Logistic	55	0.550	0.550	0.550
	SVM	55.42	0.554	0.554	0.554
	Naïve Bayes	55.42	0.562	0.554	0.539
	Random forest	56.25	0.563	0.563	0.562
	LMT	57.08	0.571	0.571	0.571
PIM	Logistic	82.08	0.823	0.821	0.821
	SVM	80.83	0.809	0.808	0.808
	Naïve Bayes	80.83	0.809	0.808	0.808
	Random forest	76.25	0.763	0.763	0.762
	LMT	83.33	0.835	0.833	0.833

For each classifier, the following metrics obtained from the tenfold cross-validation procedure are reported: validation accuracy, precision, recall, and F-score

**Table 5:** Results from four ML classification models trained on *T*-scores only for PIM and VR scales, separately

Scale	ML classifier	Accuracy (%)	Precision	Recall	*F*-score
VR	Logistic	83.33	0.833	0.833	0.833
	SVM	82.50	0.827	0.825	0.825
	Naïve Bayes	83.33	0.835	0.833	0.833
	Random forest	82.08	0.823	0.821	0.821
	LMT	83.75	0.838	0.838	0.837
PIM	Logistic	84.17	0.842	0.842	0.842
	SVM	84.17	0.843	0.842	0.841
	Naïve Bayes	84.17	0.843	0.842	0.841
	Random forest	80.42	0.804	0.804	0.804
	LMT	82.92	0.830	0.829	0.829

For each classifier, the following metrics obtained from the tenfold cross-validation procedure are reported: validation accuracy, precision, recall, and *F*-score

### Why are fakers slower only on the PIM scale?

One possible explanation for the finding that fakers were slower than honest respondents on the PIM scale, but not the VR scale, may relate to the scales’ differences in item structure. While PIM items are predominantly brief with simple syntax (e.g., “Sometimes I’m too impatient”), those of the VR scale are longer and more syntactically complex. In particular, half of the VR scale items contain a negation or a double negation (e.g., “I can honestly say that I have never met anyone I didn’t like”). Previous studies in literature have shown that negative phrases are more challenging to process than affirmative phrases (Mayo, Schul, & Burnstein, 2004). While affirmative phrases create a simple mental representation of the content, negations tend to reduce the accessibility of the information. Lower accessibility translates to longer processing times and greater errors during information processing (Kaup, Lüdtke, & Zwaan, 2006). This issue has been shown to be relevant in behavioral-based lie detection, as the cognitive load that is needed to process the negative sentences may affect both fakers and honest respondents, making it difficult to distinguish between them on the basis of RT, alone. For example, Verschuere et al. demonstrated that the use of negative sentences has a detrimental effect on accuracy in the autobiographical Implicit Association Test (aIAT), reducing accuracy from 90 to 60% (Verschuere, Prati, & Houwer, 2009).

To verify whether the lower accuracy of the VR scale in identifying faking-good respondents on the basis of temporal features was due to the presence of items with negations, we repeated the statistical analysis considering the affirmative (*n* = 5) and negative VR items (*n* = 8). In other words, the univariate analysis of variance was repeated introducing item syntax (affirmative vs. negative) as an additional within subject variable. Table 6 reports the statistically significant results of this analysis. It should be stressed that, according to the Bonferroni correction, the *p* value was divided by the number of tested variables (*n* = 4) and the significance level was set to 0.0125 (Shaffer, 1995).

**Table 6:** Significant results from the mixed ANOVA computed on RT, MD-time, vel_x_, and vel_y_ for the VR scale, introducing item syntax (affirmative vs. negative) as a variable

Variable	Effect	*F*	*p* value	$\eta_{\text{G}}^2$
RT VR	Time pressure	*F*_(1,118)_ = 29.374	3.207e^−07^	0.136 (medium)
	Items (affirmative vs. negative)	*F*_(1,118)_ = 28.104	5.431e^−07^	0.029 (small)
	Time pressure × items	*F*_(1,118)_ = 12.513	5.788e^−04^	< 0.02
MD-time VR	Time pressure	*F*_(1,118)_ = 14.048	2.774e^−04^	0.065 (small)
	Items (affirmative vs. negative)	*F*_(1,118)_ = 14.414	2.332e^−04^	0.020 (small)
	Time pressure × items	*F*_(1,118)_ = 8.405	4.463e^−03^	< 0.02
vel_x_ VR	Instructions	*F*_(1,118)_ = 9.979	0.002	< 0.02
	Instructions × items	*F*_(1,118)_ = 30.103	2.375e^−07^	0.057 (small)
vel_y_ VR	Instructions	*F*_(1,118)_ = 18.312	3.834e^−05^	0.020 (small)
	Items (affirmative vs. negative)	*F*_(1,118)_ = 24.480	2.518e^−06^	0.090 (small)
	Instructions × time pressure	*F*_(1,118)_ = 7.998	5.504e^−03^	< 0.02
	Instructions × items	*F*_(1,118)_ = 294.018	7.905e^−34^	0.388 (large)

F-score, *p*-value, and effect size ($\eta_{\text{G}}^2$) are reported for each significant effect. The *p*-value was set to 0.0125, according to the Bonferroni correction. With respect to magnitude, $\eta_{\text{G}}^2$ = 0.02 was considered indicative of a small effect, $\eta_{\text{G}}^2$ = 0.13 of a medium effect, and $\eta_{\text{G}}^2$ = 0.26 of a large effect (Cohen 1988)

The ANOVA highlighted a main effect of time pressure on both RT and MD-time. For these two variables, a significant effect of item syntax (affirmative vs. negative) was also found. Finally, statistically significant results were generated by the interaction between time pressure and item syntax, both for RT and MD-time. As concerns both vel_x_ and vel_y_, the analysis indicated a main effect of instructions and a statistically significant interaction between instructions and item syntax. A main effect of item syntax and a significant interaction between instructions and time pressure was found for vel_y_, only.

In short, for all variables except vel_x_, a statistically significant difference was found between affirmative and negative items of the VR scale. Participants were slower to respond to negative items than affirmative items (RT VR: affirmative *M* = 5349.72, SD 1981.87; negative *M* = 6121.54, SD 2834.67; MD-time VR: affirmative *M* = 3214.70, SD 1444.77; negative *M* = 3721.59, SD 2178.56); however, they moved faster along the *y*-axis when responding to negative items (vel_y_ VR: affirmative *M* = − 0.0007, SD 0.003; negative *M* = 0.0007, SD 0.002).

## Discussion

The main aim of the present research was to explore whether kinematic indicators could improve the detection of subjects implementing faking-good behavior when answering personality inventories with four choice alternatives, with and without time pressure.

### Effects of instructions

The results, first of all, indicated a successful manipulation check, as *T*-scores on the PPI-R VR scale and the PAI PIM scale were significantly higher in the faking-good condition compared to the honest condition. This result simply reflects the fact that the study instructions were correctly understood by participants: subjects instructed to fake good presented themselves in a more positive way by selecting socially desirable alternatives. This is in line with the results (Mazza, Orrù et al., 2019, Mazza, Burla et al., 2019; Roma et al., 2018; Roma, Giromini et al., 2020, Roma, Mazza et al., 2020) of prior studies investigating faking-good response styles when completing inventories with two choice alternatives (true vs. false).

The first hypothesis (H1) found support for the PIM scale but not the VR scale. For the PIM scale, respondents in the faking-good condition were slower than honest participants in terms of RT and mouse speed along the axes (vel_x_ and vel_y_), regardless of the presence or absence of time pressure. These results on the PIM scale extend previous findings (Roma et al. 2018; Roma, Giromini et al., 2020, Roma, Mazza et al., 2020), highlighting that honest respondents are faster than fakers also when answering a self-report scale with four choice alternatives (true vs. true enough vs. false enough vs. false), and not only when responding to items with dichotomous (true vs. false) alternatives. Theories for this phenomenon attribute RT differences between faking-good and honest test-takers to the fact that lying is more cognitively demanding than telling the truth (McDaniel & Timm, 1990; Verschuere, 2018) or that lying produces greater emotional arousal, due to the fear of detection (Vasilopoulos, Reilly, & Leaman, 2000). For the VR scale, we did not observe a difference in temporal variables (RT, MD-time, velocity along the *x* and *y* axes) in relation to the different instructions (honest vs. faking-good); this partially aligns with previous findings (Mazza et al., 2020), which indicated that there was no significant difference in temporal mouse dynamics (except for vel_y_) between fakers and honest test-takers. The reason why the effect of instructions on most of the temporal mouse dynamics was significant for the PIM scale but not the VR scale could be traced back to the item composition: PIM items are predominantly syntactically affirmative, whereas half of all VR items contain a negation or a double negation. Indeed, we observed that participants were slower when responding to negative items compared to affirmative ones. This result seems aligned with the psycholinguistic literature demonstrating that negative phrases have a more complex syntactic structure than affirmative phrases (Tettamanti et al., 2008) and, accordingly, they activate different areas of the brain and take more time to process (Christensen, 2009). Verschuere et al. (2009) highlighted that negative phrases limit the ability to distinguish honest from faking-good respondents on the basis of RT, alone.

### Effects of time pressure

A first result indicates a successful manipulation check: for both the PIM and the VR scales, RT and MD-time were smaller in the speeded condition; this means that participants who completed the tasks under time pressure took less time to answer compared to participants in the unspeeded condition. Surprisingly, though, time pressure did not result in any effect for vel_x_ and vel_y_. It could have been due to a failure of the manipulation check, along with the fact that we averaged all responses to items on each scale. It is possible that the effect of time pressure on velocity was present only at the beginning of the task (when the participant had just received the instructions), and disappeared as the subject proceeded with the test.

The results only partially supported the second hypothesis (H2), as no differences were found in T-scores between the speeded and unspeeded conditions for either the honest or the faking-good group. Honest respondents seemed to maintain their honesty in the speeded condition, indicating no effect of time pressure; likewise, faking-good respondents showed no significant *T*-score increase in the speeded condition, relative to the unspeeded condition. A similar finding for faking-good respondents was reported by Mazza et al., (2020) who attributed the lack of difference to a potential learning effect determined by the order in which subjects completed the tests. Specifically, the authors theorized that, when completing the task for the second time, respondents may have remembered some of the items from the first administration; such memory traces may have interfered with the effect of time pressure that has previously been observed in other studies. In a typical unspeeded condition, fakers may take longer to respond, because they must first identify the most socially desirable answer and then select this response over a more accurate self-evaluation of their personality and mental functioning. Furthermore, fakers may require additional time to estimate whether a particular answer has the potential to appear “too fake” and thus increase their risk of discovery. Significant time may be required to carry out this three-step evaluation (i.e., relating questionnaire items to one’s self, identifying the most socially desirable answer, and determining whether the question might reveal one’s faking-good behavior). Under temporal pressure, fakers may omit the final stage of the decision process, making their faking behavior more discoverable. However, in the present study, respondents who had some familiarity with the items may have been able to save sufficient time to carry out all three of the evaluation steps and, therefore, lie with less detection. Future studies should seek to verify whether the order of the tasks might indeed be associated with such a learning effect.

### ML models

To investigate the accuracy of kinematic measures in detecting faking-good participants, different ML classification models were built. This allows us to have an automatic tool that, if applied in a forensic setting, can help the clinician to make decisions about the genuineness of the examinee’s response. All classifiers showed an accuracy of around 85–86% in detecting faking-good respondents. However, further analysis revealed that just the *T*-scores and the temporal features of the PIM scale contributed to the model accuracy. In other words, in line with the statistical analysis, the kinematic measures have good classification accuracy only when the PIM scale is administered, while for the VR scale liars and truth-tellers are not distinguishable by the response times.

## Conclusions

Future research would benefit from implementing a personality questionnaire with the main purpose of detecting a faking-good response style, just as the Inventory of Problems (IOP-29), (Roma, Giromini et al., 2020, Roma, Mazza et al., 2020; Viglione, Giromini & Landis, 2017) was designed to identify a malingering or faking-bad attitude. Our study, also considering the results of previous researches on this subject (Mazza et al., 2020; Mazza, Orrù et al., 2019, Mazza, Burla et al., 2019; Roma et al., 2018; Roma, Giromini et al., 2020, Roma, Mazza et al., 2020), offers suggestions and has practical implications that could be very useful for the development of such a test, which could be particularly important in those settings in which faking-good can be expected. This test could be composed of a restricted pool of items written in a short and simple way, without negations or double negations. Items could have two or four choice alternatives, possibly inspired by the item composition of the MMPI-2 Lie scale (L) and the PAI PIM scale. Furthermore, future studies in real-life settings would help to achieve generalizability of the results outside the laboratory setting, with the aim of including behavioral features for detecting faking in personnel and forensic settings. Moreover, future studies could focus on improving converging validity by applying additional behavioral and implicit parameters and measuring these with eye-tracking and face-reading techniques.

## Supplementary Information

Below is the link to the electronic supplementary material.

Supplementary file1 (DOCX 29 KB)

## Data Availability

The datasets generated during and/or analyzed during the current study are available from the corresponding author on reasonable request.

## Footnotes

1 *T* scores (or *T*-scores) are an example of standardized scores, where the mean is equal to 50 and the standard deviation is equal to 10. They are a linear transformation of Z-scores, which have mean 0 and standard deviation 1; a T score can be obtained from a Z-score by the formula T = 50 + 10Z. T scores are convenient because scores below 0 and above 100 are virtually impossible; in fact, 99.7% of the time, a T score will lie between 20 and 80, because these limits are 3 standard deviations below and above the mean, respectively (Campbell 2013).
