# Supplementary material for: Detecting faking-good response style in personality questionnaires with four choice alternatives
Source: Psychol Res. 2021 Jan 16;85(8):3094–107. doi: 10.1007/s00426-020-01473-3 (PMC8476468; doi:10.1007/s00426-020-01473-3)
Supplement: Supplementary file 1 — Supplementary file1 (DOCX 29 KB) [file 426_2020_1473_MOESM1_ESM.docx]

**Detecting faking-good response style in personality questionnaires with four choice alternatives**

**Merylin Monaro^1†^, Cristina Mazza^2*†^, Marco Colasanti^3^, Stefano Ferracuti^3^, Graziella Orrù^4^, Alberto di Domenico^5^, Giuseppe Sartori^1^, and Paolo Roma^3^**

^1^ Department of General Psychology, University of Padova, Padova, Italy

^2^ Department of Neuroscience, Imaging and Clinical Sciences, University "G.d'Annunzio", Chieti-Pescara, Italy

^3^ Department of Human Neuroscience, Sapienza University of Rome, Rome, Italy

^4^ Department of Surgical, Medical, Molecular & Critical Area Pathology, University of Pisa, Pisa, Italy

^5^ Department of Psychological, Health and Territorial Sciences, University “G. d’Annunzio”, Chieti-Pescara, Italy

^†^ Authors contributed to the paper equally

* Correspondence: [merylin.monaro@unipd.it](mailto:merylin.monaro@unipd.it), ORCID: 0000-0001-5598-691X

**Supplementary Information**

**Details on ML classifiers parameters**

The parameters of the ML classifiers run in WEKA 3.9 software are here reported. It should be noted that these are also the default parameters automatically chosen by the software to run these algorithms.

**Logistic:**

- numDecimalPlaces -- The number of decimal places to be used for the output of numbers in the model. = 4
- batchSize -- The preferred number of instances to process if batch prediction is being performed. More or fewer instances may be provided, but this gives implementations a chance to specify a preferred batch size. = 100
- debug -- Output debug information to the console. = FALSE
- ridge -- Set the Ridge value in the log-likelihood. = 1.0E-8
- useConjugateGradientDescent -- Use conjugate gradient descent rather than BFGS updates; faster for problems with many parameters. = FALSE
- maxIts -- Maximum number of iterations to perform. = -1
- doNotCheckCapabilities -- If set, classifier capabilities are not checked before classifier is built (Use with caution to reduce runtime). = FALSE

**SVM (SMO):**

- buildCalibrationModels -- Whether to fit calibration models to the SVM's outputs (for proper probability estimates). = FALSE
- numFolds -- The number of folds for cross-validation used to generate training data for calibration models (-1 means use training data). = -1
- randomSeed -- Random number seed for the cross-validation. = 1
- c -- The complexity parameter C. = 1.0
- numDecimalPlaces -- The number of decimal places to be used for the output of numbers in the model. = 2
- batchSize -- The preferred number of instances to process if batch prediction is being performed. More or fewer instances may be provided, but this gives implementations a chance to specify a preferred batch size. = 100
- kernel -- The kernel to use. Polykernel –C 250007 -E 1.0
- checksTurnedOff -- Turns time-consuming checks off - use with caution. = FALSE
- debug -- If set to true, classifier may output additional info to the console. = FALSE
- filterType -- Determines how/if the data will be transformed. = Normalized training data
- toleranceParameter -- The tolerance parameter (shouldn't be changed). = 0.001
- calibrator -- The calibration method to use. = Logistic
- doNotCheckCapabilities -- If set, classifier capabilities are not checked before classifier is built (Use with caution to reduce runtime). = FALSE
- epsilon -- The epsilon for round-off error (shouldn't be changed). 1.0E-12

**Naïve Bayes:**

- useKernelEstimator -- Use a kernel estimator for numeric attributes rather than a normal distribution. = FALSE
- numDecimalPlaces -- The number of decimal places to be used for the output of numbers in the model. = 2
- batchSize -- The preferred number of instances to process if batch prediction is being performed. More or fewer instances may be provided, but this gives implementations a chance to specify a preferred batch size. = 100
- debug -- If set to true, classifier may output additional info to the console. = FALSE
- displayModelInOldFormat -- Use old format for model output. The old format is better when there are many class values. The new format is better when there are fewer classes and many attributes. = FALSE
- doNotCheckCapabilities -- If set, classifier capabilities are not checked before classifier is built (Use with caution to reduce runtime). = FALSE
- useSupervisedDiscretization -- Use supervised discretization to convert numeric attributes to nominal ones. = FALSE

**Logistic Model Tree (LMT):**

- splitOnResiduals -- Set splitting criterion based on the residuals of LogitBoost. There are two possible splitting criteria for LMT: the default is to use the C4.5 splitting criterion that uses information gain on the class variable. The other splitting criterion tries to improve the purity in the residuals produces when fitting the logistic regression functions. The choice of the splitting criterion does not usually affect classification accuracy much, but can produce different trees. = FALSE
- useAIC -- The AIC is used to determine when to stop LogitBoost iterations. The default is not to use AIC. = FALSE
- numDecimalPlaces -- The number of decimal places to be used for the output of coefficients. = 2
- batchSize -- The preferred number of instances to process if batch prediction is being performed. More or fewer instances may be provided, but this gives implementations a chance to specify a preferred batch size. = 100
- weightTrimBeta -- Set the beta value used for weight trimming in LogitBoost. Only instances carrying (1 - beta)% of the weight from previous iteration are used in the next iteration. Set to 0 for no weight trimming. The default value is 0. = 0.0
- doNotMakeSplitPointActualValue -- If true, the split point is not relocated to an actual data value. This can yield substantial speed-ups for large datasets with numeric attributes. = FALSE
- debug -- If set to true, classifier may output additional info to the console. = FALSE
- numBoostingIterations -- Set a fixed number of iterations for LogitBoost. If >= 0, this sets a fixed number of LogitBoost iterations that is used everywhere in the tree. If < 0, the number is cross-validated. = -1
- fastRegression -- Use heuristic that avoids cross-validating the number of Logit-Boost iterations at every node. When fitting the logistic regression functions at a node, LMT has to determine the number of LogitBoost iterations to run. Originally, this number was cross-validated at every node in the tree. To save time, this heuristic cross-validates the number only once and then uses that number at every node in the tree. Usually this does not decrease accuracy but improves runtime considerably. = TRUE
- minNumInstances -- Set the minimum number of instances at which a node is considered for splitting. The default value is 15. = 15
- doNotCheckCapabilities -- If set, classifier capabilities are not checked before classifier is built (Use with caution to reduce runtime). = FALSE
- errorOnProbabilities -- Minimize error on probabilities instead of misclassification error when cross-validating the number of LogitBoost iterations. When set, the number of LogitBoost iterations is chosen that minimizes the root mean squared error instead of the misclassification error. = FALSE
- convertNominal -- Convert all nominal attributes to binary ones before building the tree. This means that all splits in the final tree will be binary. = FALSE

**Random Forest:**

- seed -- The random number seed to be used. = 1
- storeOutOfBagPredictions -- Whether to store the out-of-bag predictions. = FALSE
- numExecutionSlots -- The number of execution slots (threads) to use for constructing the ensemble. = 1
- bagSizePercent -- Size of each bag, as a percentage of the training set size. = 100
- numDecimalPlaces -- The number of decimal places to be used for the output of numbers in the model. = 2
- batchSize -- The preferred number of instances to process if batch prediction is being performed. More or fewer instances may be provided, but this gives implementations a chance to specify a preferred batch size. = 100
- printClassifiers -- Print the individual classifiers in the output. = FALSE
- numIterations -- The number of iterations to be performed. = 100
- debug -- If set to true, classifier may output additional info to the console. = FALSE
- outputOutOfBagComplexityStatistics -- Whether to output complexity-based statistics when out-of-bag evaluation is performed. = FALSE
- breakTiesRandomly -- Break ties randomly when several attributes look equally good. = FALSE
- doNotCheckCapabilities -- If set, classifier capabilities are not checked before classifier is built (Use with caution to reduce runtime). = FALSE
- maxDepth -- The maximum depth of the tree, 0 for unlimited. = 0
- calcOutOfBag -- Whether the out-of-bag error is calculated. = FALSE
- numFeatures -- Sets the number of randomly chosen attributes. If 0, int(log_2(#predictors) + 1) is used. = 0
